# *Agrobacterium tumefaciens* Type IV and Type VI Secretion Systems Reside in Detergent-Resistant Membranes

**DOI:** 10.3389/fmicb.2021.754486

**Published:** 2021-11-25

**Authors:** Simon Czolkoss, Xenia Safronov, Sascha Rexroth, Lisa R. Knoke, Meriyem Aktas, Franz Narberhaus

**Affiliations:** ^1^Department of Microbial Biology, Ruhr University Bochum, Bochum, Germany; ^2^Department of Plant Biochemistry, Ruhr University Bochum, Bochum, Germany

**Keywords:** *Agrobacterium*, membrane organization, detergent-resistant membranes, type IV secretion system, type VI secretion system, membrane microdomains

## Abstract

Cell membranes are not homogenous but compartmentalized into lateral microdomains, which are considered as biochemical reaction centers for various physiological processes in eukaryotes and prokaryotes. Due to their special lipid and protein composition, some of these microdomains are resistant to treatment with non-ionic detergents and can be purified as detergent-resistant membranes (DRMs). Here we report the proteome of DRMs from the Gram-negative phytopathogen *Agrobacterium tumefaciens*. Using label-free liquid chromatography-tandem mass spectrometry, we identified proteins enriched in DRMs isolated under normal and virulence-mimicking growth conditions. Prominent microdomain marker proteins such as the SPFH (stomatin/prohibitin/flotillin/HflKC) proteins HflK, HflC and Atu3772, along with the protease FtsH were highly enriched in DRMs isolated under any given condition. Moreover, proteins involved in cell envelope biogenesis, transport and secretion, as well as motility- and chemotaxis-associated proteins were overrepresented in DRMs. Most strikingly, we found virulence-associated proteins such as the VirA/VirG two-component system, and the membrane-spanning type IV and type VI secretion systems enriched in DRMs. Fluorescence microscopy of the cellular localization of both secretion systems and of marker proteins was in agreement with the results from the proteomics approach. These findings suggest that virulence traits are micro-compartmentalized into functional microdomains in *A. tumefaciens*.

## Introduction

Up to one third of bacterial proteins are integral parts of the lipid bilayer and many others are tethered to the membrane via direct or indirect binding (Liu and Rost, 2001). Such membrane proteins often require a specific lipid and/or protein environment for proper function (Dowhan and Bogdanov, 2011). Eukaryotic membrane compartments, called lipid rafts are characterized by the local accumulation of cholesterol and sphingolipids (Lingwood and Simons, 2010; López and Kolter, 2010; López and Koch, 2017; Toledo et al., 2018). A number of recent studies reported similar structures in prokaryotic membranes, which differ from the rest of the membrane by a distinct lipid and protein composition (Donovan and Bramkamp, 2009). The resulting microenvironments are essential for the recruitment and assembly of membrane-associated protein complexes (López and Kolter, 2010; Bramkamp and López, 2015). Proteins of the SPFH group (stomatin/prohibitin/flotillin/HflKC) are reliable lipid raft markers in eukaryotic tissues (Browman et al., 2007). Members of this family also exist in prokaryotic organisms and are associated with detergent-resistant membranes (DRMs) (Donovan and Bramkamp, 2009; López and Kolter, 2010). Traditionally, DRMs have been separated from detergent-sensitive membranes (DSMs) by density gradient centrifugation after solubilization by various non-ionic detergents (Schuck et al., 2003; Gúzman-Flores et al., 2017). Alternatively, DRMs have successfully been isolated with a commercial kit (CelLytic^*TM*^ MEM Protein Extraction Kit) using an optimized detergent cocktail (López and Kolter, 2010; Wermser and López, 2016; Cámara-Almirón et al., 2020).

The proteome of DRMs has been identified in only a few prokaryotic model organisms, including the Gram-positive bacteria *Bacillus subtilis*, *Staphylococcus aureus*, and the Gram-negatives *Escherichia coli, Borrelia burgdorferi*, and *Pantoea* sp. YR343 (López and Kolter, 2010; Yepes et al., 2012; Toledo et al., 2015; Gúzman-Flores et al., 2017; Vijaya Kumar et al., 2020). These organisms lack the repertoire for *de novo* synthesis of cholesterol, which is a hallmark of eukaryotic lipid rafts. However, some intracellular pathogens like *B. burgdorferi* acquire cholesterol lipids from their host (Crowley et al., 2013) and integrate them into lipid raft-like microdomains that form in the inner and outer membrane (LaRocca et al., 2010, 2013; Toledo et al., 2018). The soil bacterium *B. subtilis* produces a polyisoprenoid lipid required for the proper lateral segregation of the SPFH protein FloT into DRMs suggesting that cholesterol can be functionally replaced by other sterol-like compounds in bacterial cells (López and Kolter, 2010). This assumption is supported by the identification of hopanoids (bacteriohopanetetrol cyclitol ether) in DRMs of nitrogen-fixing *Crocosphaera watsonii* (Sáenz, 2010). Staphyloxanthins have been found to physically interact with the *S. aureus* SPFH protein FloA, and loss of carotenoids in *Pantoea* sp. YR343 resulted in severe phenotypes and significantly altered membrane protein composition (Bible et al., 2016; García-Fernández et al., 2017; Vijaya Kumar et al., 2020).

Pioneering work on the membrane organization in *B. subtilis* revealed that differentiation is orchestrated by a number of proteins in distinct functional membrane microdomains (Donovan and Bramkamp, 2009; Mielich-Süss et al., 2013; Schneider et al., 2015a). Processes like sporulation and biofilm formation depend on the localization of certain proteins in a detergent-resistant membrane environment (Yepes et al., 2012). SPFH proteins presumably promote the proper localization and oligomerization (Schneider et al., 2015b). The DRM proteome from a variety of organisms suggests that other biological processes, including transport, quality control, and signaling processes are commonly associated with membrane microdomains (López and Kolter, 2010; Schneider et al., 2015a; García-Fernández et al., 2017; Vijaya Kumar et al., 2020). Recent studies established a link between membrane organization and pathogenesis, as shown by the importance of the *S. aureus* FloA in mediating infection, or by the identification of host-colonization proteins in DRMs from *B. burgdorferi* (Toledo et al., 2015; Koch et al., 2017; Mielich-Süss et al., 2017).

*Agrobacterium tumefaciens* is a Gram-negative α-proteobacterium capable of infecting primarily dicot plants via the excision and transfer of a part of its own DNA (T-DNA) into the host cell (Winans, 1992). Its pathogenic potential correlates with the integrity of several membrane-associated protein complexes, like the two-component sensor transducer system VirA/VirG and a type IV secretion system (T4SS) as well as a type VI secretion system (T6SS). The T4SS acts as a molecular needle and facilitates the translocation of the T-DNA into the plant cell upon infection (Chandran Darbari and Waksman, 2015). The integration of the T-DNA into the host chromosome results in the formation of crown gall tumors and the production of opines that are utilized as a carbon and nitrogen source by the bacteria. Interestingly, proteins of the T4SS have been reported to localize in multiple foci along the bacterial membrane (Aguilar et al., 2011). The T6SS is an antibacterial weapon beneficial for niche colonization. It delivers various effector proteins and toxins upon cell contact via a contractile shaft (Ma et al., 2014; Wu et al., 2020; Yu et al., 2021).

Given that *A. tumefaciens* produces a range of unusual lipids (see Aktas et al., 2014; Sohlenkamp and Geiger, 2016 for reviews) and harbors the genetic repertoire for the *de novo* synthesis of polyisoprenoids and other sterol-related compounds, we raised the question whether its membranes exhibit a lateral segregation and are organized into distinct functional microdomains. By identification and utilization of putative SPFH proteins as markers, we established a protocol for the isolation of DRMs from *A. tumefaciens* membranes. The proteins associated to DRMs isolated under different physiological conditions were identified by mass spectrometry. Interestingly, DRMs from virulence-induced cultures were significantly enriched with T4SS and T6SS proteins. Immunofluorescence microscopy revealed distinct T4SS and T6SS foci within the periphery of the cell. Importantly, DRM association and function of both secretion systems was irrespective of the presence of the SPFH proteins. In summary, we provide the first proteomic profile of DRMs from a plant pathogen and suggest a functional compartmentalization of *A. tumefaciens* membranes.

## Materials and Methods

### Bacterial Strains, Plasmids and Growth Condition

The strains and plasmids used in this study are listed in Supplementary Table 1. If not stated otherwise, *A. tumefaciens* and respective mutant strains were cultivated in Luria-Bertani (LB), M9 minimal medium or AB minimal medium (Schmidt-Eisenlohr et al., 1999) at 30°C supplemented with kanamycin (50 μg/ml), spectinomycin (300 μg/ml), or streptomycin (100 μg/ml) if appropriate. For virulence induction, strains were grown to an OD_600_ of 0.2 in AB minimal medium at 30°C [pH 5.5, supplemented with 1% (w/v) glucose] prior to the addition of acetosyringone (AS, Sigma-Aldrich, St. Louis, MO, United States) or DMSO to a final concentration of 0.1 mM. Cells were further incubated at 23°C for 18 h.

### Cloning and Genetic Manipulation

Recombinant DNA work was carried out according to standard methods and protocols (Sambrook and Russell, 2001). For the chromosomal integration of a 3xFLAG-tag sequence, regions upstream and downstream of the respective gene’s stop codon were amplified by PCR using the primers specified in Supplementary Table 2. The FLAG-tag coding sequence was amplified from pBO2337 (Möller et al., 2014) using the primers *3xflag_fw_bamHI* and *3xflag_rv_acc65I* inserting a TGA stop codon at the 3′ of the 3xFLAG-tag sequence. The PCR products (upstream, 3xFLAG sequence, downstream) were digested with the engineered restriction sites and subsequently ligated into pk19*mobsacB* suicide vector (Schäfer et al., 1994), resulting in plasmids pBO4701 (*atu3772*-3xFLAG), pBO4702 (*hflC*-3xFLAG), and pBO4704 (*ftsH*-3xFLAG). Chromosomal integration of plasmids was carried out as previously described by electroporation (Möller et al., 2014). Briefly, plasmids were transferred into *A. tumefaciens* via electroporation (800 Ω, 25 μF, 2 kV) and colonies screened for homologous recombination events using selective media (LB with kanamycin). Single colonies were grown for 18 h in LB without kanamycin and spread on LB plates containing 10% (w/v) sucrose. Double cross over events resulted in a sucrose tolerant and kanamycin sensitive phenotype. Putative mutants containing the 3xFLAG-tag sequence *in frame* with the target gene on the chromosome were verified by colony PCR. For the construction of mutagenesis plasmids to delete genes encoding for SPFH proteins, 400–500 bp fragments up- and downstream of *atu3772, hflK*, and *hflC* were amplified using the primer pairs specified in Supplementary Table 2. PCR product pairs were digested with *Pst*I, ligated and cloned into pK19*mobsacB* suicide vector using the restriction sites for *Eco*RI and *Hin*dIII resulting in the plasmid pBO3709 (for *atu3772* deletion), pBO3710 (for *hflC* deletion) and pBO3711 (for *hflK* deletion). Mutant construction was carried out as described before (Czolkoss et al., 2016). Double (*hflKC*) and triple (*hflKC*/*atu3772*) deletion was achieved using respective *hflK* single and *hflKC* double mutant strains as a background for mutagenesis.

### Membrane Preparation and Detergent-Resistant Membranes Fractionation

Cultures were grown to early stationary phase in LB or AB minimal medium as described above and OD_600_ was adjusted to 4. Cells were harvested at 4°C and supernatant was discarded. Pellets were washed and resuspended in 700 μl 50 mM Tris-HCl (pH 7.4). Next, 1 mM PMSF and 1.5 mg/ml lysozyme were added and cells were further incubated for 90 min at 4°C. Disruption was achieved by ultrasonication using a VialTweeter instrument (Hielscher, Teltow, Germany). Cell debris was removed by centrifugation at 20.000 × *g* at 4°C for 15 min. Membranes were pelleted via ultracentrifugation at 320.000 × *g* for 90 min at 4°C. Two hundred microliter of supernatant were transferred to a fresh microcentrifuge tube and stored for further analysis (cytosolic samples, “C”). Membrane pellets were resolved in 600 μl Lysis and Separation Buffer from the CelLytic^*TM*^ MEM Protein Extraction Kit (Sigma-Aldrich, St. Louis, MO, United States) containing 6 μl of the provided protease inhibitor cocktail. The lysate was centrifuged for 15 min at 20.000 × *g* and 4°C. Subsequently, 100 μl of supernatant were transferred to a fresh microcentrifuge tube and stored for further analysis (membrane samples, “M”). Phase-separation between DSMs and DRMs was carried out following the manufactures’ instructions. Ultimately, 100 μl of each fraction were stored for further analysis.

### SDS-PAGE and Western Blotting

For sodium dodecyl sulfate-polyacrylamide gel electrophoresis (SDS-PAGE) and subsequent Western blotting of the membrane preparation samples, proteins from equal volumes were precipitated using chloroform and methanol. Briefly, samples were mixed with 4x volumes of methanol and vortexed. 1x volume of chloroform was added and samples were again vortexed before the addition of 3x volumes *milliQ* grade water. Samples were centrifuged at 20.000 × *g* for 6 min at room temperature. Upper layer was removed and 3x volumes of methanol were added. Samples were again centrifuged at 20.000 × g for 6 min and methanol was evaporated in a SpeedVac system. Protein pellets were resolved in 1x SDS sample buffer, containing 1 M Tris (pH 6.5), 50% glycerol (vol/vol), 10% SDS (wt/vol), 0.5% bromophenol blue (wt/vol) and 5% β-mercaptoethanol (vol/vol). Equal volumes of fractions were analyzed by SDS-PAGE and Western blot analysis. FLAG-tagged proteins were detected using monoclonal ANTI-3XFLAG M2 antibody (Sigma-Aldrich, St. Louis, MO, United States) and secondary goat anti-mouse IgG (H + L)-HRP conjugate (Bio-Rad, Hercules, CA, United States). VirB5, VirB9, TssI, and TssH were detected using protein-specific antibodies (rabbit) and secondary goat anti-rabbit IgG (H + L)-HRP conjugate (Bio-Rad, Hercules, CA, United States). Protein signals were visualized using Luminata Forte Western HRP substrate (Merck-Millipore, Billerica, MA, United States). For signal detection, a ChemiImager Ready system (Alpha Innotec, Kasendorf, Germany) was used.

### Fluorescence Microscopy

For immunofluorescence, cells were cultivated as described above in LB or AB minimal medium to early stationary phase, harvested and adjusted to an OD_600_ of 2 in PBS buffer. Cells were fixed in 2.7% paraformaldehyde and 0.01% glutaraldehyde for 30 min at 4°C. Cells were washed in PBS and GTE buffer containing 50 mM glucose, 10 mM EDTA, and 20 mM Tris. Permeabilization of the membrane was achieved by incubation in PBS buffer containing 5 mM EDTA and 5 mg/ml lysozyme at 4°C for 5 min followed by PBS washing and incubation in PBS containing 0.1% Triton X-100 for 5 min at room temperature. Triton X-100 was removed by PBS washing and cells were blocked with PBST [PBS containing 0.1% tween 20 (vol/vol)] containing 3% BSA (wt/vol) for 1 h at room temperature. Cells were again washed in PBST and incubated with primary antibody solution in PBS, 0.1% BSA for 1 h at 30°C. After washing with PBST, cells were incubated with secondary antibody solution in PBST for 1 h at 30°C. Finally, cells were washed three times in PBST, resuspended in 200 μl PBS and mounted onto agar pads containing 0.5% low-melting agarose. Cells were imaged with an Olympus BX51 microscope using a U-UCD8 condenser and an UPlanSApo 100XO objective and images were taken with a CC12 digital color camera (all components by Olympus, Hamburg, Germany).

### Mass Spectrometry and Data Analysis

Protein and LC-MS/MS analysis was carried out as previously described (Plohnke et al., 2014, 2015). Briefly, proteins were concentrated to a single band in SDS gels by terminating electrophoresis when the migration front reached the interface between stacking and separating gel. Protein bands were cut out and digested with trypsin (Rexroth et al., 2003). Peptides were extracted from the gel using 50% (vol/vol) acetonitrile and 1% (vol/vol) formic acid prior to lyophilization. For LC-MS/MS analysis, peptides were re-solubilzed in 0.1% (vol/vol) formic acid. For reversed-phase chromatography a gradient of solvent A [0.1% (vol/vol) formic acid] and solvent B [99.9% (vol/vol) acetonitrile and 0.1% (vol/vol) formic acid] was used. MS-analysis was carried out using a Thermo LTQ Orbitrap XL mass spectrometer in duty cycle consisting of one 400–2000 m/z FT-MS and four MS/MS LTQ scans. MS and MS/MS data were acquired using Xcalibur (all by Thermo Fisher Scientific, Waltham, MA, United States). Each sample consisted of three independent biological replicates. Obtained LC-MS/MS spectra were analyzed using the Sequest algorithm (Eng et al., 1994). Peptides were matched against UniProt database of *A. tumefaciens* for protein identification. Proteins were quantified using spectral counting and normalized spectral abundance factors (NSAF) when identified by at least one unique peptide in two of three replicates (Plohnke et al., 2015). Proteins were classified as significantly enriched in DRMs or DSMs according to Student’s *t*-test on a significance level of 95% if their ratio was greater than 1.5 or lower than 0.7. Automated localization prediction of proteins found in DRM and DSM fractions was performed using the CELLO2GO algorithm (*E*-value set to 0.005) (Yu et al., 2014). Protein groups were identified and quantified with information from the UniProt and KEGG database and the BlastKOALA annotation tool (Kanehisa et al., 2016). The mass spectrometry proteomics data have been deposited to the ProteomeXchange Consortium via the PRIDE partner repository with the dataset identifier PXD028782 (Perez-Riverol et al., 2019).

### Lipid Analysis

Phospholipids of *A. tumefaciens* strains were isolated according to Bligh and Dyer and analyzed as previously described (Bligh and Dyer, 1959; Czolkoss et al., 2016). For two-dimensional TLC (2D-TLC), mixtures of chloroform:methanol:water (65:25:4) and chloroform:methanol:acetic acid:water (90:15:10:3.5) were used as running solvents for first and second dimension, respectively. For the visualization of the lipids, plates were charred after CuSO_4_-treatment at 180°C

### Biofilm Formation Assay

Biofilm formation of *A. tumefaciens* strains was quantified using a crystal violet staining assay as described elsewhere with slight modifications (Knoke et al., 2020). Briefly, 1 ml of M9 minimal medium in a 12-well microtiter plate (Sarstedt, Nümbrecht, Germany) was inoculated with overnight cultures to an OD_600_ of 0.5. Plates were incubated for 3 days at 15°C. After 24, 48, and 72 h, 50 μL crystal violet (0.5%) were added and the plates were further incubated for 10 min at room temperature with gentle rocking. Wells were carefully washed three times with 1.5 ml water and the dye was eluted from the organic material with 1 ml 100% ethanol. Biofilm was quantified by measuring the absorbance at 570 nm.

### Motility Assay

Swimming motility of *A. tumefaciens* was analyzed by spotting 3 μl of stationary phase cultures on M9 minimal medium soft agar plates [0.5% agar (wt/vol)]. Plates were incubated for 24 h at 30°C and swarm diameters were measured.

### Virulence Assay

Functionality of the T4SS was monitored using the AGROBEST assay as described previously (Wu et al., 2014; Groenewold et al., 2019). Briefly, *Arabidopsis* seeds were sterilized and germinated in MS medium for 7 days at 22°C in a 16 h/8 h light/dark period prior to infection. *A. tumefaciens* wild type and SPFH mutant strains were transformed with pBISN1 harboring the *gusA*-intron, cultivated in AB minimal medium for virulence induction as described above and resuspended to an OD_600_ of 0.02 in infection medium (50 ml MS medium, 50 ml AB medium, 200 μM AS). *Arabidopsis* seedlings were incubated with *A. tumefaciens* solutions for 3 days as described above and measured for transient GUS expression by staining with 5-bromo-4-chloro-3-indolyl glucuronide (X-Gluc) for 3 h at 37°C.

### Type VI Secretion Assay

T6SS-dependent secretion of TssD (Hcp) was evaluated as described before with minor modifications (Wu et al., 2008). Briefly, *A. tumefaciens* cultures grown in AB minimal medium at 30°C for 6 h were harvested by centrifugation and total protein samples were prepared using 1x SDS sample buffer. Supernatants harboring the secreted proteins were filtered through a 0.45 μm membrane and concentrated by TCA precipitation. Protein pellets were resuspended in 10 μl of 1 M Tris buffer and 10 μl 2x SDS sample buffer. Equal volumes of total protein samples (T) and supernatant-derived samples (S) were analyzed by SDS-PAGE and Western detection of TssD using a protein-specific antibody as described above.

## Results

### *Agrobacterium tumefaciens* Stomatin/Prohibitin/Flotillin/HflKC Proteins Are Co-purified With Detergent-Resistant Membranes

**FIGURE 1:**
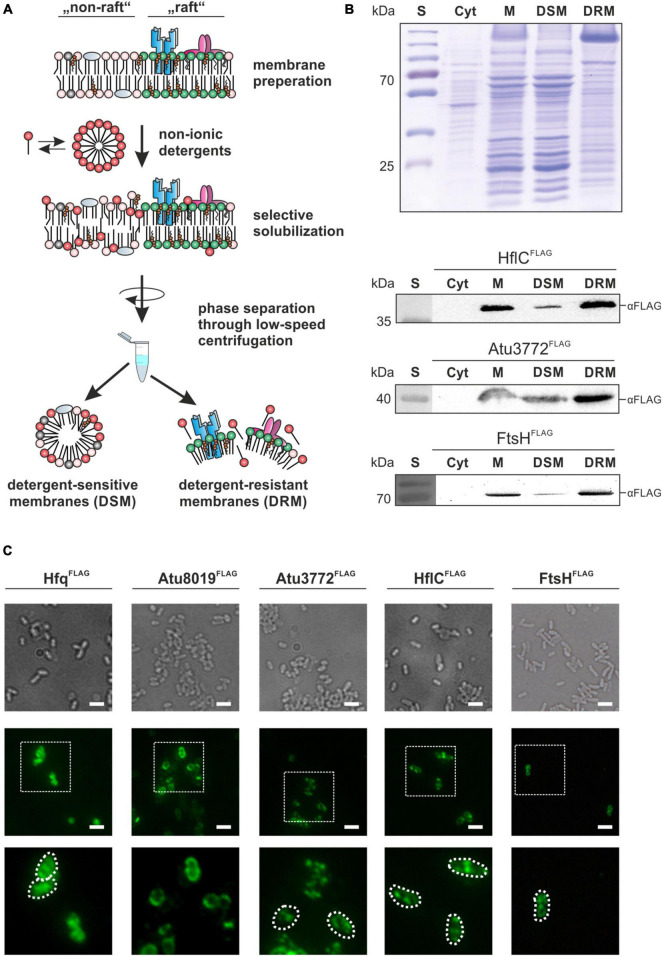
Stomatin/prohibitin/flotillin/HflKC (SPFH) proteins are co-purified with detergent-resistant membranes and form discrete membrane foci. **(A)** Schematic depiction of the DRM-purification workflow. Isolated membranes are selectively solubilized by detergent-treatment and separated via low-speed centrifugation. **(B)** SDS-PAGE and Western analysis of membrane fractions from LB-grown strains. After ultracentrifugation of disrupted cells, samples from the supernatant containing the cytoplasmic fraction were obtained (Cyt). The membrane pellet (M) was resuspended in Lysis and Separation Buffer (Sigma-Aldrich, St. Louis, MO, United States) before phase separation by centrifugation and incubation yielded detergent-sensitive membranes (DSM) and detergent-resistant membranes (DRM). Presence of HflC, Atu3772 and FtsH in the different samples was analyzed by biochemical fractionation of membranes from corresponding FLAG-tagged strains as described above. Proteins were detected using FLAG-tag specific antibodies. S, standard. **(C)** Immunofluorescence analysis revealed a spotty localization pattern of Atu3772, HflC, and FtsH in the membrane of strains expressing the corresponding genes as FLAG-tagged variants from their native gene locus. Hfq and Atu8019 served as controls for a cytosolic or homogenous membrane localization, respectively. Protein localization was monitored using FLAG-tag specific primary antibodies followed by fluorescent secondary antibodies (AlexaFluor^488^). Bar represents 5 μm.

To explore the presence and protein composition of membrane microdomains in *A. tumefaciens*, we established a DRM isolation protocol using the CelLytic^*TM*^ MEM Protein Extraction Kit (Sigma-Aldrich, St. Louis, MO, United States). This kit has successfully been applied to bacterial model organisms like *B. subtilis, B. anthracis*, and *Staphylococcus aureus* (López and Kolter, 2010; Somani et al., 2016) and enables rapid purification of DRMs without tedious sucrose density centrifugation (Wermser and López, 2016). Our protocol is based on an initial membrane preparation followed by selective solubilization (by a mixture of non-ionic detergents) and subsequent low-speed centrifugation to achieve phase separation between DSMs and DRMs (Figure 1A). This protocol is suitable for the isolation of DRMs from the inner membrane (IM) and outer membrane (OM), which is important since microdomains are found in both membranes (Toledo et al., 2018). SDS-PAGE analysis of the DRM and DSM fractions from cells grown in LB medium revealed a distinct and reproducible protein pattern with some proteins – especially in the higher molecular range – enriched in DRMs, while others were depleted (Figure 1B).

Proteins from the SPFH (stomatin/prohibitin/flotillin/HflKC) family are commonly co-purified with DRMs (Browman et al., 2007) making them suitable markers for the validation of our membrane fractionation protocol. We searched for putative SPFH homologs in *A. tumefaciens* and identified three candidates. The genes *atu2044* (*hflC*) and *atu2045* (*hflK*) encode putative modulators of the FtsH protease, and *atu3772* codes for a protein with high homology to *E. coli* QmcA, another FtsH interaction partner (Supplementary Figure 1A) (Chiba et al., 2006). The *hflC* and *hflK* genes form a bicistronic operon on the circular chromosome. Atu3772 is encoded on the linear chromosome immediately upstream of *atu3773*, coding for an NfeD (nodulation formation efficiency D)-like protein. SPFH domains presumably are important for interaction with the membrane. In HflC, HflK and Atu3772 they are followed by predicted coiled-coil regions involved in protein oligomerization (Rivera-Milla et al., 2006). Importantly, the three *Agrobacterium* SPFH proteins lack the C-terminal flotillin domain (InterPro: IPR031905) found in some prokaryotic flotillin homologs, such as *B. subtilis* FloT (Supplementary Figure 1B).

We generated strains expressing *atu3772, hflC*, and *ftsH* as FLAG-tagged variants from their native gene locus and validated protein production by Western blot analysis using FLAG-tag specific antibodies (Supplementary Figure 1C). The signals corresponded to the calculated molecular masses (Atu3772^FLAG^: 40.5 kDa; HflC^FLAG^: 37.5 kDa; FtsH^FLAG^: 73.5 kDa). Protein levels of HflC and Atu3772 were irrespective of growth whereas the FtsH signal diminished in late stationary phase. This might be explained by the loss of the C-terminal FLAG-tag due to the self-processing activity of FtsH in late growth phases (Akiyama, 1999).

All three FLAG-tagged proteins in the engineered strains were exclusively found in the membrane, and not in the cytoplasmic fraction (Figure 1B) demonstrating that the tag does not interfere with proper membrane localization of these proteins. Moreover, HflC^FLAG^, Atu3772^FLAG^, and FtsH^FLAG^ showed a substantial enrichment in the DRM fraction, which is in good agreement with data from *B. subtilis*, *S. aureus*, and *B. burgdorferi*, and therefore validates the practicability of our purification protocol (López and Kolter, 2010; Yepes et al., 2012; Toledo et al., 2015).

### *In vivo* Localization of Stomatin/Prohibitin/Flotillin/HflKC Proteins

Given that *A. tumefaciens* SPFH proteins were co-purified with DRMs, which are thought to originate from a spatial membrane segregation into distinct domains, one would expect a patchy localization of these proteins *in vivo*. To test this assumption, we used an immunofluorescence-based approach to visualize Atu3772, HflC, and FtsH thanks to their FLAG-tag. Additionally, strains producing Hfq or Atu8019 as FLAG-tagged variants were analyzed as controls for a cytosolic or uniform membrane localization, respectively (Figure 1C; Knoke et al., 2020). The two SPFH proteins Atu3772 and HflC, as well as FtsH were detected in multiple and distinct fluorescent foci after treatment with FLAG-antibodies (mouse) followed by incubation with AlexaFluor 488 goat-anti-mouse secondary antibodies. A wild-type control strain did not exhibit any fluorescence signals under the same conditions (data not shown).

### The Proteome of Detergent-Resistant Membranes From *A. tumefaciens* Grown in Rich Medium

The findings described above prompted us to identify the protein composition of *A. tumefaciens* DRMs. Proteins residing in DRMs from cells grown in LB medium were identified by an LC-MS/MS-based approach, and peptides were matched against the *A. tumefaciens* UniProt database. For statistical parameters, see the materials and methods section. In total, 73 proteins were classified as “DRMs only” (n = 53) or “strongly enriched in DRMs” (*n* = 20). Eighty nine proteins localized to DSMs, and 49 thereof were classified as “DSMs only” (see Supplementary Table 3 for a full list). A list with selected proteins discussed in more detail below is included in Table 1. Localization prediction of the proteins identified in DRMs suggests a substantial enrichment of inner and outer membrane proteins (Supplementary Figure 2A). A number of annotated “cytoplasmic” proteins resided in the DSM fractions, which might be the result of proteins dynamically interacting with the membrane, thus being included in the initial membrane fraction prior to detergent treatment. This assumption is corroborated by the identification of components of the division machinery (FtsA), the respiratory chain (NuoE, NuoF, NuoG), and substrate binding proteins of ABC transporter systems in DSMs (Table 1).

**TABLE 1:** Protein composition of DRMs and DSMs from cell grown in LB medium.

	**Name**	**Gene**	**UniProtID**	**Function**	**Ratio DRM/DSM**	***p*-value**
SPFH proteins	HflC	*atu2044*	A9CIC9	Modulator of FtsH	10.34	0.004
	HflK	*atu2045*	Q7CY01	Modulator of FtsH	10.46	0.019
	Atu3772 (QmcA)	*atu3772*	A9CFM5	Probable modulator of FtsH	5.03	0.003
	FtsH	*atu3710*	Q7CT50	ATP-dependent zinc metalloprotease	2.92	0.042
DRM- associated	PpiD	*atu1686*	A9CIS0	Peptidyl-prolyl *cis-trans* isomerase	6.24	0.013
	SecY	*atu1927*	Q7CY83	Protein translocase subunit SecY	DRM only	0.096
	SecD	*atu1562*	Q7CYZ3	Protein-export membrane protein SecD	DRM only	0.177
	YidC	*atu0384*	Q8UIB3	Membrane protein insertase YidC	DRM only	0.028
	TssL (ImpK)	*atu4333*	Q7CUN4	OmpA-like porin	DRM only	0.015
	AvhB9	*atu5170*	Q7D3R4	Type IV secretion protein AvhB9	DRM only	0.001
	AvhB10	*atu5171*	Q7D3R3	Type IV secretion protein AvhB10	DRM only	0.001
	AvhB11	*atu5172*	Q7D3R2	Type IV secretion protein AvhB11	DRM only	0.016
	MotB	*atu3746*	Q7CT75	Flagellar motor protein	DRM only	0.036
	TatA	*atu1706*	Q8UEP9	Sec-independent protein translocase protein TatA	DRM only	0.194
	TatB	*atu1705*	Q8UEQ0	Sec-independent protein translocase protein TatB	DRM only	0.201
	TatC	*atu1704*	A9CIR3	Sec-independent protein translocase protein TatC	DRM only	0.185
	Atu0251 (MbfA)	*atu0251*	A9CKH9	Unknown	DRM only	0.180
	SipF	*atu1034*	A9CJK6	Signal peptidase I	DRM only	0.002
	Atu3773	*atu3773*	A9CFM6	Unknown	DRM only	0.234
	Atu3744 (MsbA)	*atu3744*	A9CFL3	ABC transporter, nucleotide binding/ATPase protein	DRM only	0.037
	LptD	*atu1106*	A9CJH2	LPS-assembly protein LptD	DRM only	0.076
	Atu2749 (LptE)	*atu2749*	A9CHE8	LPS-assembly lipoprotein LptE	DRM only	0.088
	Atu3203	*atu3203*	Q7CRV9	RND multidrug efflux membrane permease	DRM only	0.045
	Atu4063	*atu4063*	Q7CTZ7	O-linked GlcNAc transferase	DRM only	0.037
	Atu0027	*atu0027*	A9CKS6	Two component sensor kinase	DRM only	0.047
	Atu4400	*atu4400*	A9CGK7	Ferrienterobactin-like protein	DRM only	0.005
	Atu4524	*atu4524*	A9CGS6	ABC transporter, membrane spanning protein	DRM only	0.002
	HppA	*atu1174*	Q8UG67	K(+)-insensitive pyrophosphate-energized proton pump	DRM only	0.035
	ExoP	*atu1239*	A9CJC4	Exopolysaccharide production protein	DRM only	0.019
DSM-associated	NuoG	*atu1276*	Q7CZL6	NADH ubiquinone oxidoreductase chain G	0.17	0.002
	NuoE	*atu1274*	A9CJA7	NADH ubiquinone oxidoreductase chain E	0.13	0.011
	NuoF	*atu1275*	A9CJA6	NADH ubiquinone oxidoreductase chain F	DSM only	0.008
	FtsA	*atu2087*	P0A331	Cell division protein FtsA	DSM only	0.035
	DctP	*atu5268*	A9CLG5	ABC transporter, substrate binding protein	0.33	0.031
	Atu2281	*atu2281*	Q7CXG0	ABC transporter, substrate binding protein	DSM only	0.040

*Stomatin/prohibitin/flotillin/HflKC proteins and FtsH are among the most prominent proteins identified in DRMs. Additional proteins discussed in the manuscript are indicated as DRM-associated or DSM-associated. The ratios were calculated by the quotient of the respective normalized spectral abundance factors of the proteins. Protein annotation according to UniProt database.*

**FIGURE 2:**
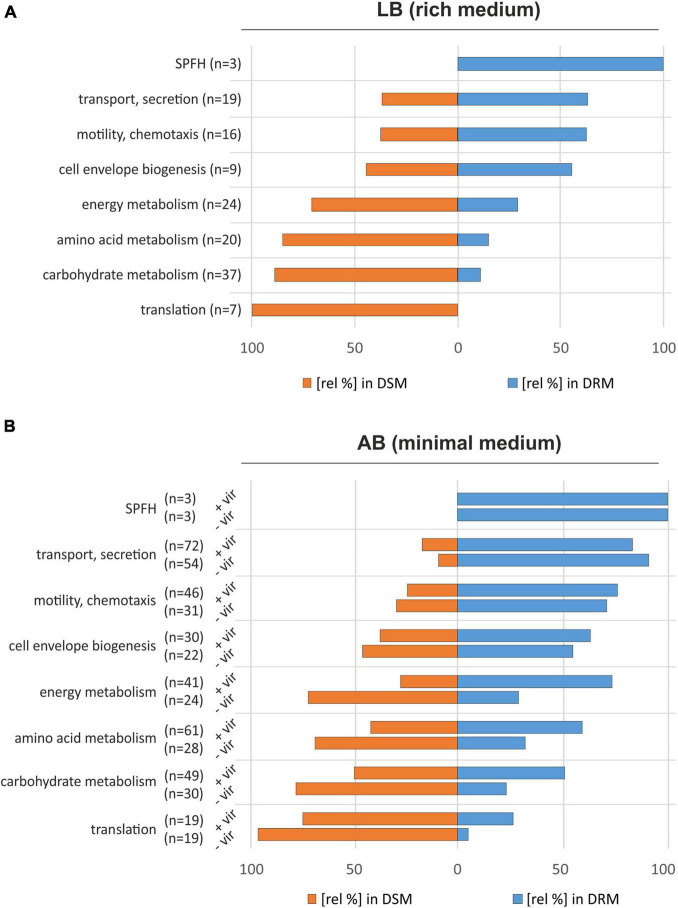
Proteomic comparison of *A. tumefaciens* DRMs and DSMs from cultures grown in LB medium **(A)** and AB minimal medium **(B)**. Protein functions were assigned using GO terms provided by the UniProt database combined with information from the KEGG database (Kanehisa et al., 2016). The bars indicate the relative distribution of proteins from the different categories in the DRM and DSM fractions. –vir: non-induced cultures; +vir: virulence-induced cultures.

To attribute biological functions to the identified proteins, we used information from the UniProt and KEGG databases and screened for selected functional groups using the BlastKOALA webservice (Kanehisa et al., 2016). The SPFH proteins HflK, HflC, and Atu3772 were manually assigned as an individual group (SPFH) with all three members being most abundant in DRMs (Figure 2A and Table 1). Other strongly overrepresented proteins in DRMs were involved in cell envelope biogenesis (e.g., MsbA, LptDE, ExoP), transport and secretion (e.g., TssL, AvhB9-11), or assigned to motility- and chemotaxis-related processes (e.g., MotB, Atu0027). Interestingly, several FtsH substrates and interaction partners like PpiD, SecY, SecD, and YidC were also found in DRMs (Table 1). PpiD is a membrane-associated chaperone that interacts with the Sec translocon and is degraded by FtsH, at least in *E. coli* (Sachelaru et al., 2014; Bittner et al., 2015). SecY and SecD are additional FtsH substrates in *E. coli* (Akiyama et al., 1996; Arends et al., 2016; Bittner et al., 2017), and a direct interaction between SecY and a bacterial SPFH protein was demonstrated in *B. subtilis* (Bach and Bramkamp, 2013). The FtsH interaction partner YidC mediates the insertion of multimeric protein complexes into the membrane (van Bloois et al., 2008). In contrast, proteins enriched with DSMs were involved in various metabolic processes, e.g., energy, amino acid, or carbohydrate metabolism and translation (Figure 2A).

The strong overrepresentation of proteins involved in transport or secretion processes, as well as the identification of FtsH along with its potential modulators (HflK, HflC, and Atu3772) and subtrates (PpiD, SecY, SecD) in the DRM fractions suggests a functional partitioning between detergent-soluble and non-soluble membrane domains within the plasma membranes of *A. tumefaciens*.

### Virulence-Associated T4SS and T6SS Are Localized in Detergent-Resistant Membranes

*Agrobacterium tumefaciens* deploys two bacterial secretion systems. The expression of genes coding for a T4SS, which delivers T-DNA and effector proteins into the plant cell, is induced in response to environmental signals such as phenolic compounds released from wounded plant cells and acidic milieus (Chen and Winans, 1991). The acid-induced T6SS provides a competitive advantage against other bacteria during plant colonization (Wu et al., 2012; Ma et al., 2014). Since the T6SS component TssL and proteins of the T4SS-like AvhB system (AvhB9-11) were identified in the DRM fractions from LB-grown cultures (Table 1), we hypothesized that further structural components of both the T4SS and the T6SS might reside in DRMs isolated from virulence-induced *A. tumefaciens* cells. We thus cultivated *A. tumefaciens* in AB minimal medium (pH 5.5) supplemented with acetosyringone (AS) to induce the virulence cascade. Membranes of virulence-induced (+vir) and non-induced (–vir) cultures were purified, separated into DRMs and DSMs, and subjected to LC-MS/MS. Using the criteria defined above for quality control of the raw data, 278 proteins were found to be enriched in the DRM fractions from non-induced cultures. 130 of them were classified as “DRM only” (see Supplementary Table 3 for a complete list). Thirty-one of the 142 proteins identified in the DSM fraction were absent in the DRM fraction and therefore categorized as “DSM only.” Under virulence-induced conditions, 453 proteins were overrepresented in DRMs (235 “DRM only”) and 156 in the DSM fractions (50 “DSM only”). It has been noted previously that different cellular activities under different physiological conditions can account for differences in the DRM proteome (Vijaya Kumar et al., 2020). This might explain the higher number of proteins identified from cells grown in AB compared to LB medium. Medium-dependent differences are supported by a large overlap of 128 proteins in the DRM proteome of *A. tumefaciens* grown in AB medium with and without acetosyringone (Supplementary Figure 2B). In total, 27 proteins were found in the DRM fractions under all three conditions (Supplementary Figure 2B and Table 2).

**TABLE 2:** The DRM “core” proteome.

	**Name**	**Gene**	**UniProtID**	**Function**
Marker	HflC	*atu2044*	A9CIC9	Modulator of FtsH
	HflK	*atu2045*	Q7CY01	Modulator of FtsH
	Atu3772 (QmcA)*	*atu3772*	A9CFM5	Probable modulator of FtsH
	FtsH*	*atu3710*	Q7CT50	ATP-dependent zinc metalloprotease
DRM-associated	YidC	*atu0384*	Q8UIB3	Membrane protein insertase YidC
	OrdL	*atu0601*	A9CK55	Oxidoreductase
	Atu0650	*atu0650*	A9CK37	Unknown
	Atu0994	*atu0994*	A9CJL8	Electron transfer flavoprotein-ubiquinone oxidoreductase
	Sipf	*atu1034*	A9CJK6	Signal peptidase I
	MltG	*atu1099*	Q7CZZ6	Endolytic murein transglycosylase
	HppA	*atu1174*	Q8UG67	K(+)-insensitive pyrophosphate-energized proton pump
	PlsX	*atu1178*	Q8UG63	Phosphate acyltransferase (Acyl-ACP phosphotransacylase)
	MrcA	*atu1341*	A9CJ77	DD-transpeptidase
	Atu1357	*atu1357*	Q7CZF4	Unknown
	FixG	*atu1530*	A9CIY9	Nitrogen fixation protein FixG
	Nah	*atu1574*	A9CIX0	Salicylate hydroxylase
	KefB	*atu1693*	A9CIR7	Glutathione-regulated potassium-efflux system protein
	Atu2279	*atu2279*	A9CI32	ABC transporter, nucleotide binding/ATPase protein
	Atu2284	*atu2284*	A9CI31	Unknown
	Atu2550	*atu2550*	Q7CWT1	RND multidrug efflux membrane permease
	Atu2710	*atu2710*	Q7CWF5	Peptidase_M48 domain-containing protein
	Atu3507	*atu3507*	Q7CSM9	SH3b domain-containing protein
	Atu3546	*atu3546*	A9CFD4	Unknown
	MotB	*atu3746*	Q7CT75	Flagellar motor protein
	Atu3797	*atu3797*	A9CFN9	HlyD family secretion protein
	Gcd	*atu4135*	A9CG47	Glucose dehydrogenase
	Atu5089	*atu5089*	Q7D3Y0	Unknown
	RcdB	*atu5091*	Q7D3X8	Curdlan synthesis protein
	Atu8095	*atu8095*	Q8U4W5	Unknown

*List of proteins associated to DRMs under all tested conditions (LB, AB [–vir], AB [+vir]).*

*Atu3772 and FtsH were added although they did not meet the stringent criteria to be statistically significant under [–vir] conditions (*p*-values of 0.08 and 0.12, respectively, instead of the defined threshold of 0.05). Protein annotation according to UniProt database.

The DRM enrichment of proteins belonging to functional groups like “cell envelope biogenesis” or “transport and secretion” in AB medium was consistent with the results from LB-grown cultures (Figure 2B). As expected, HflC, HflK, Atu3772, and FtsH showed a clear preference for the DRM fraction and did not respond to AS treatment (Figure 2B and Table 3). The absolute number of proteins involved in transport/secretion and motility/chemotaxis increased in both DRMs and DSMs upon virulence induction while their proportional distribution between both fractions remained largely unaffected. The percentage of many proteins involved in “housekeeping” metabolic processes (energy, amino acid, carbohydrate metabolism, and translation) increased after AS treatment in DRMs. This might be explained by profound VirA/G-induced metabolic restructuring in anticipation of opines, that are produced and secreted by transfected plants. Presumably due to their low abundance, we were unable to identify many of the proteins (derived from the *noc-*region of the Ti-plasmid) responsible for the uptake and metabolism of nopaline in *A. tumefaciens* C58 (Subramoni et al., 2014).

**TABLE 3:** Type IV secretion system (T4SS) and type VI secretion systems (T6SS) are enriched in *A. tumefaciens* DRMs.

					**Virulence-induction**				
	**Name**	**Gene**	**UniprotID**	**Function**	**Membrane**	**DSM**	**DRM**	**ratio DRM/DSM [+vir]**	***p*-value**
marker	HflC	*atu2044*	A9CIC9	Modulator of FtsH	±	±	±	8.23	0.002
	HflK	*atu2045*	Q7CY01	Modulator of FtsH	±	±	±	3.53	0.024
	Atu3772 (QmcA)	*atu3772*	A9CFM5	Probable modulator of FtsH	±	±	±	2.83	0.003
	FtsH	*atu3710*	Q7CT50	ATP-dependent zinc metalloprotease	±	±	±	2.92	0.002
*vir* (T4SS)	VirK	*atu6156*	Q7CNV8	VirA/G regulated protein	n.i.	n.i.	n.i.	–	–
	VirA	*atu6166*	P18540	Two component chemoreceptor	+	±	Only + vir	DRM only	0.027
	VirG	*atu6178*	P07545	Two component transcriptional activator	+	+	+	3.07	0.017
	VirB2	*atu6168*	P17792	Pore/pilus like structure	Only + vir	n.i.	Only + vir	–	0.423
	VirB3	*atu6169*	P17793	Pore/pilus like structure	Only + vir	n.i.	Only + vir	DRM only	0.170
	VirB4	*atu6170*	P17794	Pore/pilus like structure	+	+	+	5.37	0.000
	VirB5	*atu6171*	P17795	Pore/pilus like structure	Only + vir	Only + vir	Only + vir	DRM only	0.271
	VirB6	*atu6172*	P17796	Pore/pilus like structure	n.i.	n.i.	Only + vir	–	0.423
	VirB7	*atu6173*	P17797	Pore/pilus like structure	Only + vir	n.i.	+	DRM only	0.038
	VirB8	*atu6174*	P17798	Pore/pilus like structure	+	Only + vir	Only + vir	11.04	0.039
	VirB9	*atu6175*	P17799	Pore/pilus like structure	+	+	+	9.70	0.009
	VirB10	*atu6176*	P17800	Pore/pilus like structure	+	+	+	5.89	0.005
	VirB11	*atu6177*	P07169	Pore/pilus like structure	+	Only + vir	+	15.77	0.079
	VirC2	*atu6179*	P07166	DNA binding	Only + vir	Only + vir	+	30.44	0.004
	VirC1	*atu6180*	P07165	DNA binding	Only + vir	Only + vir	Only + vir	2.48	0.076
	VirD1	*atu6181*	P18591	DNA binding, endonuclease	Only + vir	Only + vir	Only + vir	1.39	0.297
	VirD2	*atu6182*	P18592	DNA binding, endonuclease	Only + vir	Only + vir	+	1.78	0.165
	VirD3	*atu6183*	P18593	Unknown	+	+	+	0.16	0.232
	VirD4	*atu6184*	P18594	Coupling factor	+	+	+	7.69	0.068
	VirD5	*atu6185*	A9CL19	Secreted into plant cell	±	n.i.	±	–	0.423
	VirE1	*atu6189*	P08063	Chaperone like protein for VirE2	n.i.	n.i.	n.i.	–	–
	VirE2	*atu6190*	P08062	DNA binding	+	+	+	1.00	0.985
	VirE3	*atu6188*	P08061	Unknown	n.i.	n.i.	Only – vir	–	–
	VirF	*atu6154*	Q7D2D9	F-box protein	n.i.	n.i.	n.i.	–	–
*imp* (T6SS)	TagE (ImpN)	*atu4330*	Q7CUN1	Serine/threonine protein kinase	+	±	±	DRM only	0.037
	TagF (ImpM)*	*atu4331*	Q7CUN2	Serine/threonine phosphoprotein	±	–	+	4.50	0.010
	TssM (ImpL)	*atu4332*	A9CGF9	IcmF family protein	±	±	±	3.39	0.029
	TssL (ImpK)	*atu4333*	Q7CUN4	OmpA-like porin	±	+	±	1.61	0.313
	TssK (ImpJ)	*atu4334*	A9CGG0	Unknown	±	±	±	0.47	0.018
	TagH (ImpI)	*atu4335*	A9CGG1	Putative fha-domain protein	±	–	±	4.86	0.010
	TssG (ImpH)	*atu4336*	A9CGG2	Unknown	+	n.i.	+	DRM only	0.092
	TssG (ImpG)	*atu4337*	A9CGG3	Unknown	+	+	+	1.63	0.218
	TssF (ImpF)	*atu4338*	Q7CUN9	Unknown	Only – vir	n.i.	±	–	0.423
	TagJ (ImpE)*	*atu4339*	Q7CUP0	Unknown	+	–	+	DRM only	0.021
	TssC_40_ (ImpD)	*atu4340*	A9CGG4	Unknown	Only + vir	Only + vir	Only + vir	–	0.730
	TssC_41_ (ImpC)	*atu4341*	A9CGG5	Pore/pilus like structure	±	+	+	1.79	0.009
	TssB (ImpB)	*atu4342*	A9CGG6	Pore/pilus like structure	+	+	+	1.99	0.317
	TssA (ImpA)	*atu4343*	A9CGG7	Unknown	±	–	±	30.40	0.062
*hcp* (T6SS)	TssH (ClpV)	*atu4344*	Q7CUP5	ATP-dependent Clp protease	±	–	±	7.13	0.013
	TssD (Hcp)	*atu4345*	A9CGG8	Hcp, needle complex	–	–	–	14.44	0.067
	Atu4346*	*atu4346*	A9CGG9	Unknown	n.i.	–	Only – vir	–	0.423
	Atu4347*	*atu4347*	Q7CUP8	Peptidoglycan amidase	±	–	±	2.19	0.027
	Tssl-1 (VgrG)	*atu4348*	A9CGH0	VgrG protein	±	±	±	1.68	0.019
	Atu4349*	*atu4349*	Q7CUQ0	Unknown	±	±	+	0.95	0.751
	Atu4350*	*atu4350*	A9CGH1	Nuclease	+	±	+	1.18	0.490
	Atu4351*	*atu4351*	Q7CUQ2	Unknown	±	±	+	0.98	0.862
	Atu4352*	*atu4352*	A9CGH2	Unknown	Only + vir	Only + vir	Only – vir	–	0.423

*Stomatin/prohibitin/flotillin/HflKC proteins and FtsH do not respond to virulence-induction but are significantly enriched within DRMs. Proteins deduced from the *vir* operon are highly enriched in DRMs of virulence-induced cells. The T6SS consists of the *imp* and the *hcp* operon. Proteins encoded by the *imp* operon show a significant enrichment within the DRMs. For the *hcp* operon, only TssH, TssD and Atu4347 display a DRM enrichment. Proteins marked with an asterisk are dispensable for T6SS-dependent secretion according to Ma et al. (2014). Protein annotation according to UniProt database and Lin et al. (2013) and Ma et al. (2014). + or –: increased or decreased relative protein amount after virulence-induction; ±: no significant change in relative protein amount; n.i.: protein not identified; Only +/− vir: protein was only identified after/without virulence induction.*

Predictions of cellular localization of proteins in DRMs and DSMs were similar to those of LB medium, indicating the robustness of our protocol for the isolation of membrane proteins along with DRMs (Supplementary Figure 2A).

Strikingly, many of the T4SS-associated proteins were found to be highly enriched (e.g., VirB4, VirB8-10) or identified exclusively in the DRMs fractions (e.g., VirB7) of virulence-induced cultures (Table 3). Transcription of the *vir* operon is controlled by the two-component system VirA/VirG, which senses phenolic compounds and activates transcription of *vir* genes encoded on the Ti-plasmid. Notably, both proteins were either exclusively found (the sensor kinase VirA) or strongly enriched (the response regulator VirG) within DRM fractions of AS-treated cultures (Table 3). As expected, many of the VirA/VirG-regulated proteins were identified only after virulence induction (e.g., VirB2-3. VirB5, VirB7, VirC1-2, VirD1-2).

The T6SS machinery is encoded in two different operons (*imp* and *hcp*), which are both activated under acidic conditions via transcriptional regulation by ChvG/ChvI (Wu et al., 2012). Interestingly, T6SS functionality decreases in the presence of phenolic compounds, which presumably results from reduced amounts of tube forming TssD (Wu et al., 2012; Lin et al., 2013). Consistent with these reports, we observed that the relative amounts of TssD were lowered when AS was added to the medium (Table 3). Importantly, several of the T6SS proteins from both operons were strongly overrepresented in DRM fractions (e.g., TagFH, TssH, TssM TagE, TssC_40_). These data clearly demonstrated the preference of both T4SS and T6SS for a detergent-resistant membrane environment, suggesting that lateral membrane organization into domains of distinct physicochemical parameters might be beneficial for infection and interspecies competition.

**FIGURE 3:**
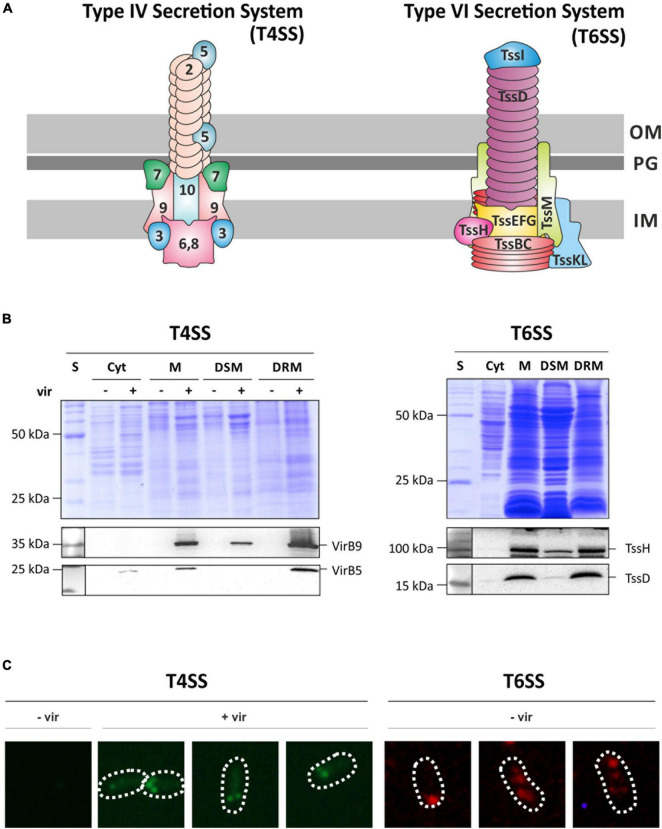
Structural components of the T4SS and the T6SS are co-purified with DRMs and form discrete membrane foci. **(A)** Schematic representation of both secretory complexes (modified after Leiman et al., 2009; Chandran Darbari and Waksman, 2015). Vir-proteins are numbered according to their protein names. T6SS-associated proteins are named according to the *tss* nomenclature (Lin et al., 2013). OM, outer membrane; PG, peptidoglycan; IM, inner membrane. **(B)** SDS-PAGE and Western analysis of fractionated membrane samples from non-induced (–vir) or virulence-induced (+vir) cultures. After blotting, membranes were cut horizontally and the presence of VirB5, VirB9, TssH, and TssD in the different fractions was analyzed by immunodetection using specific antibodies as described above. S, standard; Cyt, cytosolic fraction; M, membrane fraction. **(C)** Localization of the T4SS was analyzed by detecting VirB5 using protein-specific antisera followed by fluorescent-probed secondary antibodies (AlexaFluor^488^). Under non-induced conditions (–vir), VirB5 was not detected. After induction of the virulence-cascade (+vir), multiple fluorescence foci along the perimeter of the cell were detected. Localization of the T6SS was monitored by the detection of TssH in AB minimal medium under non-virulent conditions as described above.

To validate the localization of the T4SS and T6SS to restricted membrane domains by an independent approach, we tracked the localization of selected components of these secretion systems by specific antibodies (Figure 3A). Following SDS-PAGE and Western blotting, antisera against VirB5 and VirB9 as components of the T4SS and against the T6SS components TssH (ClpV) and TssD (Hcp) were used (Figure 3B). Consistent with the MS analysis, both Vir proteins were detected at the calculated sizes (VirB9: 32 kDa; VirB5: 23 kDa) in total membrane fractions (M) and in DRMs of virulence-induced cultures. Only small amounts of VirB9 were found in DSMs from AS-treated cultures, and VirB5 was not at all detectable in DSM fractions. TssH and TssD were detected at the calculated molecular masses of 96 and 17 kDa, respectively, and were clearly enriched in DRMs.

We further investigated the spatial distribution of the T4SS and T6SS *in vivo* using an immunofluorescence-based approach (Figure 3C). The T4SS was detected via the minor pilus core component VirB5 and the T6SS via the ATPase subunit TssH using monoclonal primary antibodies followed by AlexaFluor secondary antibody treatment. As expected, VirB5 was not detected under non-induced conditions (–vir). After virulence induction (+vir), several fluorescence foci appeared at the periphery of the cell. VirB5 has a role during pilus assembly and primarily localizes to the tip of the pilus (Aly and Baron, 2007). The T6SS component TssH was also found in multiple spots along the perimeter of the cell when grown in AB minimal medium (–vir). In summary, these findings provide evidence that the delivery of DNA and effector proteins via both secretion systems is restricted to specific sites in the bacterial membrane that confer detergent resistance to the embedded proteins.

### Association of T4SS and T6SS to DRMs Does Not Depend on the Presence of Stomatin/Prohibitin/Flotillin/HflKC Proteins

**FIGURE 4:**
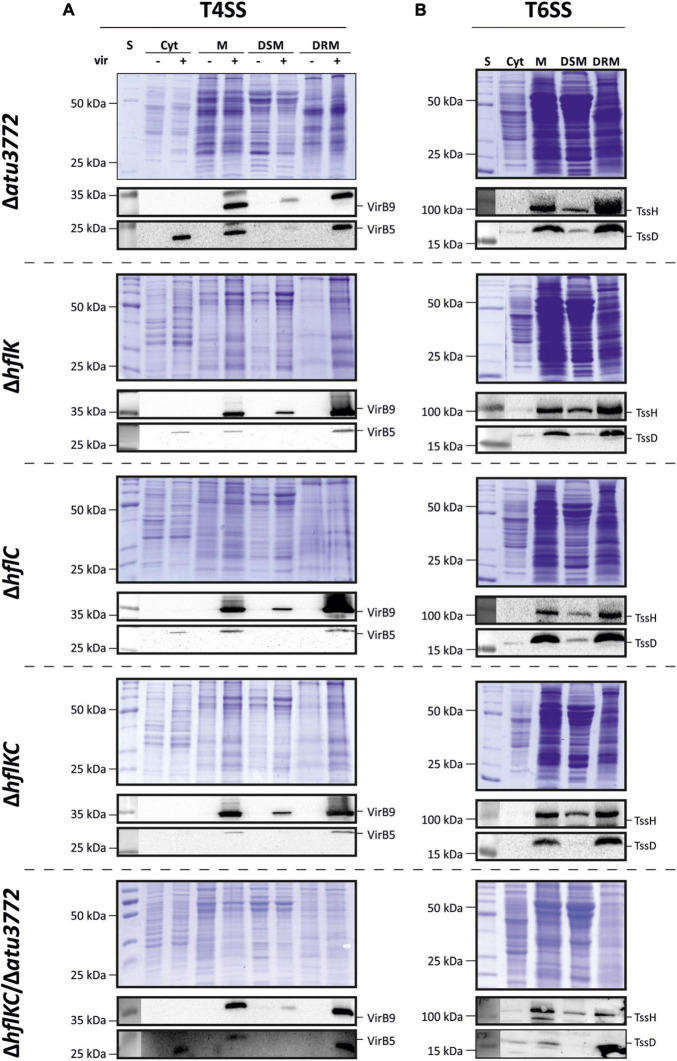
Localization of the secretory complexes T4SS and T6SS to DRMs does not depend on SPFH proteins. Membranes from non-induced and virulence-induced cultures were fractionated into DRMs and DSMs before proteins were precipitated and analyzed via SDS-PAGE and immunodetection as described above. Presence of VirB5 and VirB9 **(A)** and TssH and TssD **(B)** in the different fractions was analyzed using protein-specific antibodies.

Because SPFH proteins are hallmarks of both prokaryotic and eukaryotic DRMs, we asked whether the localization of *A. tumefaciens* T4SS and T6SS in DRMs is dependent on these proteins. Corresponding single-, double-, and triple-mutant strains were constructed and analyzed for various phenotypes that might be associated with an altered membrane organization. All mutants showed wild type-like growth, biofilm formation and motility (Supplementary Figure 3A). Lipid profiles from SPFH mutant strains were indistinguishable from the wild type featuring phosphatidylglycerol (PG), phosphatidylethanolamine (PE), monomethyl-PE (MMPE), phosphatidylcholine (PC), ornithine lipids (OL), and cardiolipin (CL) in comparable amounts (Supplementary Figure 3B). Importantly, T4SS-mediated transformation of *Arabidopsis* seedlings and T6SS-mediated secretion of TssD were not impaired in SPFH mutants compared with the wild type (Supplementary Figures 3C,D). Accordingly, the enrichment of VirB5 and VirB9 (T4SS; Figure 4A) as well as TssH and TssD (T6SS; Figure 4B) in DRM fractions purified from the different mutant strains was not altered. Cumulatively, these results suggest that Atu3772, HflK, and HflC are dispensable for DRM localization and functionality of both the T4SS and the T6SS.

## Discussion

Compartmentalization is thought to be a universal strategy to fine tune the assembly, activity and stability of membrane proteins (López and Koch, 2017). Despite some shortcomings, detergent extraction is widely used to study membrane domains. Diverse membrane proteins involved in signal transduction, cell division and membrane transport processes are enriched in DRMs, and the function of several of these proteins depends on their subcellular localization in microdomains (Yepes et al., 2012; López and Koch, 2017). Although DRM localization might not necessarily reflect membrane substructures in living cells, the separation of membranes into DRMs and DSMs is suited to provide valuable insights into membrane organization. Our study combines proteomics, immunodetection and fluorescence microscopy and presents evidence for the partitioning of *Agrobacterium* membrane proteins into distinct entities. Several interesting aspects of our findings are discussed below.

(i) In support of a valid fractionation approach, three SPFH proteins and the FtsH protease were enriched in DRMs isolated under any tested condition (Table 2). They came along with several proteins known to be FtsH substrates in *E. coli*. Some of these proteins participate in protein translocation (PpiD, SecY, SecD), thus taking part in the FtsH-mediated quality control of aberrant proteins (Bittner et al., 2017). These findings are in good agreement with the proteomic characterization of lipid rafts in the human pathogen *B. burgdorferi*. Toledo and colleagues demonstrated the strong enrichment of FtsH, HflK, and HflC as well as SecD in DRMs (Toledo et al., 2015). The Gram-positive model bacterium *B. subtilis* lacks close homologs of HflK and HflC. Instead, FtsH function and activity depends on the presence of the two SPFH proteins FloT and FloA (formerly YqfA). Together with FtsH, they are part of the protein cargo of *B. subtilis* DRMs (López and Kolter, 2010; Yepes et al., 2012) suggesting that the co-localization of the FtsH protease with its modulators in DRMs is a common feature. Atu3772, the third SPFH protein in *A. tumefaciens*, was also found in DRMs. It is homologous to the *E. coli* QmcA protein, which physically interacts with FtsH and, together with YbbJ, acts as a multicopy suppressor of cells lacking FtsH (Chiba et al., 2006). YbbJ is encoded in the same operon as *qmcA* and belongs to the NfeD protein family. The occurrence of NfeD/SPFH gene pairs is widespread in prokaryotic genomes and probably linked to membrane protein homeostasis (Kuwahara et al., 2008). Atu3773/Atu3772, an equivalent protein pair in *A. tumefaciens*, was identified in DRMs under all tested conditions (Table 1 and Supplementary Table 3) suggesting a role of DRMs in membrane protein quality control.

(ii) Gram-negative bacteria are characterized by an outer membrane composed of lipopolysaccharides (LPS) in the outer leaflet. MsbA is an ABC transporter responsible for flipping LPS across the cytoplasmic membrane (Polissi and Georgopoulos, 1996). LPS molecules are then transferred to the LPS transport (Lpt) machinery, which facilitates their translocation to the outer leaflet of the outer membrane (Okuda et al., 2016). MsbA (Atu3744) belongs to the *A. tumefaciens* DRM proteome in complex and minimal medium (Table 1 and Supplementary Table 3). Interestingly, each of the Lpt proteins spanning the inner membrane (LptBCFG) or outer membrane (LptDE) were identified under at least one culture condition in DRMs (Supplementary Table 3). The periplasmic protein LptA connects the inner- and outer membrane-spanning components via binding to LptC and LptD (Bowyer et al., 2011) and was found in DSMs (Supplementary Table 3). LPS are important virulence factors of Gram-negative pathogenic bacteria and are ingested by macrophages upon infection (Molinaro et al., 2015). Intriguingly, *Brucella abortus* LPS have been shown to induce macrodomains at the cell surface of macrophages (Lapaque et al., 2006) and experiments on artificial lipid vesicles suggested that LPS have an effect on membrane segregation similar to that of cholesterol (Henning et al., 2009). These findings raise the question of whether lateral lipid domains exist in both the IM and OM of Gram-negative bacteria. Toledo et al. (2018) recently reported lipid microdomains with distinct characteristics in both membranes of *B. burgdorferi*. Moreover, DRM enrichment of several microdomain marker proteins (FtsH, HflK, HflC, YidC, SecD) was identical for total membrane-derived DRMs and IM-derived DRMs (Toledo et al., 2015, 2018). Given these reports and our results that several OM proteins were present in DRMs and DSMs (Figures 3, 4), we assume that DRMs are present in both membranes of *A. tumefaciens*. However, since the solubilization efficiencies of both membranes with certain detergents might differ, the analysis needs to be refined in the future. Of note, the lipid profile of DRMs resembles that of total membranes, containing anionic PG and CL and zwitterionic PE, MMPE, OL, and PC, the latter being slightly reduced compared to total membranes (Supplementary Figure 4).

(iii) DRMs have been implicated in membrane transport processes and our data fully support this notion. In addition to several ABC transporter proteins, we identified multiple RND multidrug efflux systems in DRMs (Supplementary Table 3). These systems facilitate the export of toxic substances across the cellular membranes and thus are involved in resistance to antibiotics. Drug transporters have been identified in DRMs from the pathogenic yeast *Candida albicans* suggesting that membrane microdomains are important for detoxification processes (Pasrija et al., 2008). Apart from the Sec machinery, we identified the Tat system in DRMs (Table 1 and Supplementary Table 3). In contrast to the Sec system, the Tat pathway is used for the translocation of already folded proteins across the membrane (Natale et al., 2008). Taken together, these findings support the concept that membrane microdomains serve as platforms for the import or export of various molecules into or out of the cell.

(iv) Probably the most intriguing finding of our study was the identification of numerous virulence-associated proteins in the DRM fraction. The function of these proteins ranges from the perception of the wounded plant (VirAG two-component system) to the actual T-DNA transfer machinery. T4SS systems are widespread in bacterial pathogens and deliver effector proteins and/or DNA into adjacent bacterial or host cells (see Chandran Darbari and Waksman, 2015; Grohmann et al., 2018 for reviews). All proteins of the *A. tumefaciens* VirB-pilus core-complex (VirB3-5, VirB7-11), together with VirC1, VirC2, VirD2, and VirD4 were co-purified with DRMs from virulence-induced cultures. VirD2, together with VirC1/2 participates in binding and translocation of the T-DNA to the VirB/D4 translocation apparatus, where it is transferred to target cells (Lu et al., 2009; Padavannil et al., 2014). Furthermore, the DRM association of the VirAG system emphasizes the importance of a lateral segregation in concentrating signaling and transport/secretion to defined membrane environments. Another connection between T4SS function and membrane organization was recently reported in the human pathogen *Helicobacter pylori*. Here, T4SS-mediated translocation of the extracellular effector protein CagA was significantly reduced in cells lacking the SPFH protein HP0248, presumably due to impaired cholesterol acquisition (Hutton et al., 2017). The virulence-associated type VII secretion system (T7SS) of *S. aureus* is part of the DRM protein cargo and partially co-localizes with the SPFH protein FloA (Mielich-Süss et al., 2017).

The cellular localization and number of functional T4SS clusters within *A. tumefaciens* membranes is debated. GFP fusions of proteins from the translocation core complex (VirB3/4, VirB8, VirB11) or VirD4 are primarily detected at a single spot at one of the cell poles (Christie, 2004; Mossey et al., 2010; Das and Das, 2014). However, studies from the Zambryski lab using both GFP fusions and direct immunofluorescence imaging demonstrated the arrangement of several T4SS structural components in a helical pattern around the bacterial perimeter (Aguilar et al., 2010, 2011). Our data support the idea of multiple T4SS sites as demonstrated by the visualization of native VirB5 in the membranes of virulence-induced cultures (Figure 3).

(v) SPFH proteins and flotillin homologs are generally believed to promote the correct assembly of membrane proteins into microcompartments (López and Koch, 2017). Due to this scaffolding activity, the *S. aureus* flotillin FloA was proposed to be an attractive target for antimicrobial compounds (García-Fernández et al., 2017; Koch et al., 2017; Mielich-Süss et al., 2017). However, super-resolution microscopy questioned the role of flotillins in the generation of functional multiprotein clusters (Dempwolff et al., 2016). Moreover, T7SS-mediated secretion of effector proteins is reduced but still detectable in the absence of FloA in *S. aureus* (Mielich-Süss et al., 2017), and reports on the significance of FloA/FloT for sporulation and biofilm formation in *B. subtilis* are conflicting (López and Kolter, 2010; Devi et al., 2015; Schneider et al., 2015b). We found that the three SPFH proteins of *A. tumefaciens* were neither required for DRM localization and function of the T4SS and T6SS nor for motility and biofilm formation. The physiological role of bacterial SPFH proteins presumably is multifaceted and many phenotypes are not immediately apparent. *E. coli* mutants lacking the SPFH protein YqiK are unaffected under many membrane-associated stress conditions, but show altered swimming behavior compared to wild type (Padilla-Vaca et al., 2019). It is thus possible that Atu3772, HflK and HflC have a role under yet unknown conditions. Among others, these observations add a new perspective to this debate and suggest that the exact function of SPFH proteins in bacterial membranes is incompletely understood and needs to be carefully evaluated in an array of Gram-positive and Gram-negative species.

## Data Availability Statement

The datasets presented in this study can be found in online repositories. The names of the repository/repositories and accession number(s) can be found below: PRIDE database, PXD028782.

## Author Contributions

FN supervised the project. MA and FN conceived and planned the experiments. SC and XS performed the experiments. SR carried out the mass spectrometric analysis. SC wrote the manuscript with support of FN and MA. All the authors contributed to the article and approved the submitted version.

## Conflict of Interest

The authors declare that the research was conducted in the absence of any commercial or financial relationships that could be construed as a potential conflict of interest.

## Publisher’s Note

All claims expressed in this article are solely those of the authors and do not necessarily represent those of their affiliated organizations, or those of the publisher, the editors and the reviewers. Any product that may be evaluated in this article, or claim that may be made by its manufacturer, is not guaranteed or endorsed by the publisher.
