# Supplementary material for: Agrobacterium tumefaciens Type IV and Type VI Secretion Systems Reside in Detergent-Resistant Membranes
Source: Front Microbiol. 2021 Nov 25;12:754486. doi: 10.3389/fmicb.2021.754486 (PMC8656257; doi:10.3389/fmicb.2021.754486)
Supplement: Supplementary file 1 [file Data_Sheet_1.pdf]

Supplementary Material

Supplementary Tables

Supplementary Table 1: Bacterial strains and plasmids used in this study.

Strain or plasmid	Relevant characteristics[a]	Reference or source
**Strain**		
Agrobacterium tumefaciens C58	Wild type	C. Baron, Montreal, Canada
A. tumefaciens Δ*atu3772*	Wild type derivative, deletion of the *atu3772* gene	This study
A. tumefaciens Δ*hflK*	Wild type derivative, deletion of the *hflK* (*atu2045*) gene	This study
A. tumefaciens Δ*hflC*	Wild type derivative, deletion of the *hflC* (*atu2044*) gene	This study
A. tumefaciens Δ*hflKC*	Wild type derivative, deletion of the *hflK* and *hflC* genes	This study
A. tumefaciens Δ*hflKC*/Δ*atu3772*	Wild type derivative, deletion of the *hflK*, *hflC* and *atu3772* genes	This study
A. tumefaciens atu8019^{FLAG}	Chromosomal integration of 3XFLAG tag sequence at *atu8019* 3' end	(Knoke et al., 2020)
A. tumefaciens hfq^{FLAG}	Chromosomal integration of 3XFLAG tag sequence at *hfq* 3' end	(Möller et al., 2014)
A. tumefaciens atu3772^{FLAG}	Chromosomal integration of 3XFLAG tag sequence at *atu3772* 3' end	This study
A. tumefaciens hflC^{FLAG}	Chromosomal integration of 3XFLAG tag sequence at *hflC* 3' end	This study
A. tumefaciens ftsH^{FLAG}	Chromosomal integration of 3XFLAG tag sequence at *ftsH* (*atu3710*) 3' end	This study
Escherichia coli DH5α	Cloning host	(Hanahan, 1983)
**Plasmid**		
pYPRUB168	Amp^R; *lacZα,* pUC18 derivative, vector used for cloning	(Hoffmann et al., 2015)
pK19*mobsacB*	Km^R; suicide vector used for mutant construction	(Schäfer et al., 1994)
pBO2337	Derivative of pK19*mobsacB* carrying upstream region including *hfq* ORF with 3xFLAG tag sequence and downstream region	(Möller et al., 2014)
pBO3709	Derivative of pK19*mobsacB* carrying up- and downstream region of *atu3772*	This study

pBO3710	Derivative of pK19*mobsacB* carrying up- and downstream region of *atu2044*	This study
pBO3711	Derivative of pK19*mobsacB* carrying up- and downstream region of *atu2045*	This study
pBO4701	Derivative of pK19*mobsacB* carrying upstream region of *atu3772* 3' end with 3xFLAG tag sequence and downstream region	This study
pBO4702	Derivative of pK19*mobsacB* carrying upstream region of *atu2044* 3' end with 3xFLAG tag sequence and downstream region	This study
pBO4704	Derivative of pK19*mobsacB* carrying upstream region of *atu3710* 3' end with 3xFLAG tag sequence and downstream region	This study

[a]*Ap: ampicillin; Km: kanamycin;* Str: streptomycin; Spc: spectinomycin

Supplementary Table 2: Oligonucleotides used in this study.

Oligonucleotide	Sequence (5‘ - 3‘)[a]
**Mutant construction**	
atu3772-up_ecoRI_fw	AAAGAATTCAGCCCTATACCGACAAGACCG
atu3772-up_pstI_rv	AAACTGCAGGCCCAATGCAATCATCACTTTCC
atu3772-down_pstI_fwI	AAACTGCAGTCATCGGAGAAGTAACGATGTTGC
atu3772-down_hindIII_rv	AAAAAGCTTGAGTTGCGGTGCGGCCGACAAGG
hflC-up_ecoRI_fw	AAAGAATTCATGCCTTCGACGAAGTGCAGCG
hflC-up_pstI_rv	AAACTGCAGAAGACGGTTACCCATATCAGTTGCC
hflC-down_pstI_fw	AAACTGCAGGCGCCGGCGAACTGATAGCGG
hflC-down_hindIII_rv	AAAAAGCTTGAGCAGCGGCTCGGCAAGATCAG
hflK-up_ecoRI_fw	AAAGAATTCGGACGAGGTTTTTGTCGCCGGC
hflK-up_pstI_rv	AAACTGCAGATTGCTCCAGGGCATCAATACC
hflK-down_pstI_fw	AAACTGCAGGCAGGGAGGCAACTGATATGG
hflk-down_hindIII_rv	AAAAAGCTTCGATGCGTCGAGACGGGTGCG
**3XFLAG tag integration**	
3xflag_fw_bamHI	AAAAGGATCCGACTACAAAGACCATGACGGTG
3xflag_rv_acc65I	AAAAGGTACCTCATTTATCGTCGTCATCTTTGTAG
atu3772up_fw_pst	AAAACTGCAGGCACAGGTGCTGGA
atu3772up_rv_bamHI	AAAAGGATCCCTTCTCCGATGAAGAG
atu3772down_fw_acc65I	AAAAGGTACCCGATGTTGCAACGGCT
atu3772down_rv_ecoRI	AAAAGAATTCGCGGTGCGGCCGACAAG
hflC_up_fw_pstI	AAAACTGCAGGCGCATGAAGTCGGAACGTCTTGC
hflC_up_rv_bamHI	AAAAGGATCCGTTCGCCGGCGCTGGCG

hflC_down_fw_acc65I	AAAAGGTACCTAGCGGATCGAATGATTTTGGA
hflC_down_rv_ecoRI	AAAAGAATTCCACCACCGCATCGAGCAG
ftsH_up_fw_hindIII	AAAAAAGCTTGCGCCATGGTTACGC
ftsH_up_rv_bamHI	AAAAGGATCCATGCGGCTGCGGCTC
ftsH_down_fw_acc65I	AAAAGGTACCTCGCCTGCGCGAACGA
ftsH_down_rv_ecoRI	AAAAGAATTCGCGAGGCGGAGATCATC

[a]restriction sites are underlined

Supplementary Table 3: List of proteins identiefied in DRMs and DSMs from *A. tumefaciens* grown under different conditions. Proteins were quantified using spectral counting and normalized spectral abundance factors (NSAF) when identified by at least one unique peptide in two of three replicates. Proteins were classified as significantly enriched in DRMs or DSMs according to Student's *t*-test on a significance level of 95% if their ratio [DRM/DSM] was greater than 1.5 or lower than 0.7.

Supplementary Figures

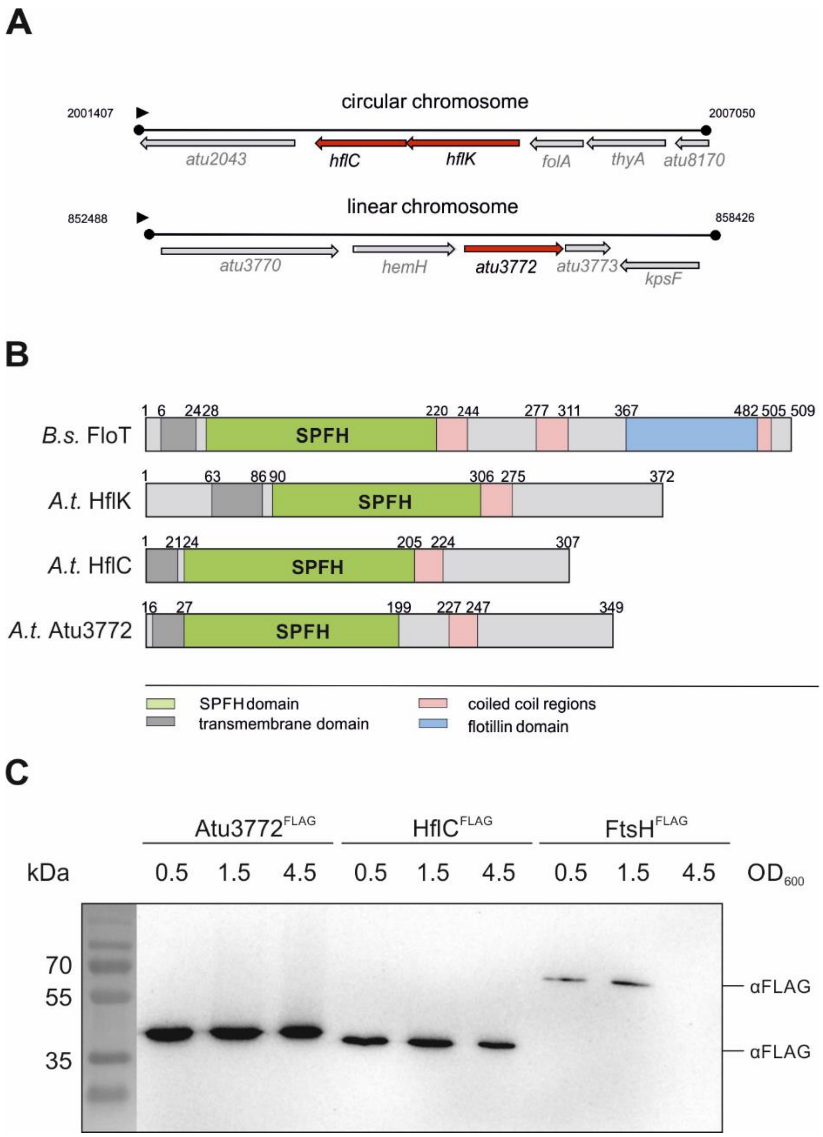

Fig. S1: *A. tumefaciens* encodes three SPFH proteins. (A) Chromosomal organization of the SPFH coding genes. *hflK* and *hflC* form a putative bicistronic operon and are encoded on the circular chromosome. *atu3772* is located on the linear chromosome. *folA*: dihydrofolate reductase; *thyA*: thymidylate synthase; *hemH*: ferrochelatase; *kpsF*: capsule expression protein. (B) Domain organization of *A. tumefaciens* SPFH proteins HflK, HflC and Atu3772 in comparison with the extensively studied FloT from *B. subtilis*. Domains have been annotated according to *Pfam* database. (C) Detection of *A. tumefaciens* DRM-marker proteins in different growth-phases. Strains expressing *atu3772, hflC* and *ftsH* as FLAG-tagged variants from the authentic gene locus were grown in LB medium and samples were taken at indicated optical densities of the respective cultures. Proteins in cellular extracts were separated by SDS-PAGE. After Western blotting, HflC, Atu3772 and FtsH were detected by FLAG-tag-specific antibodies.

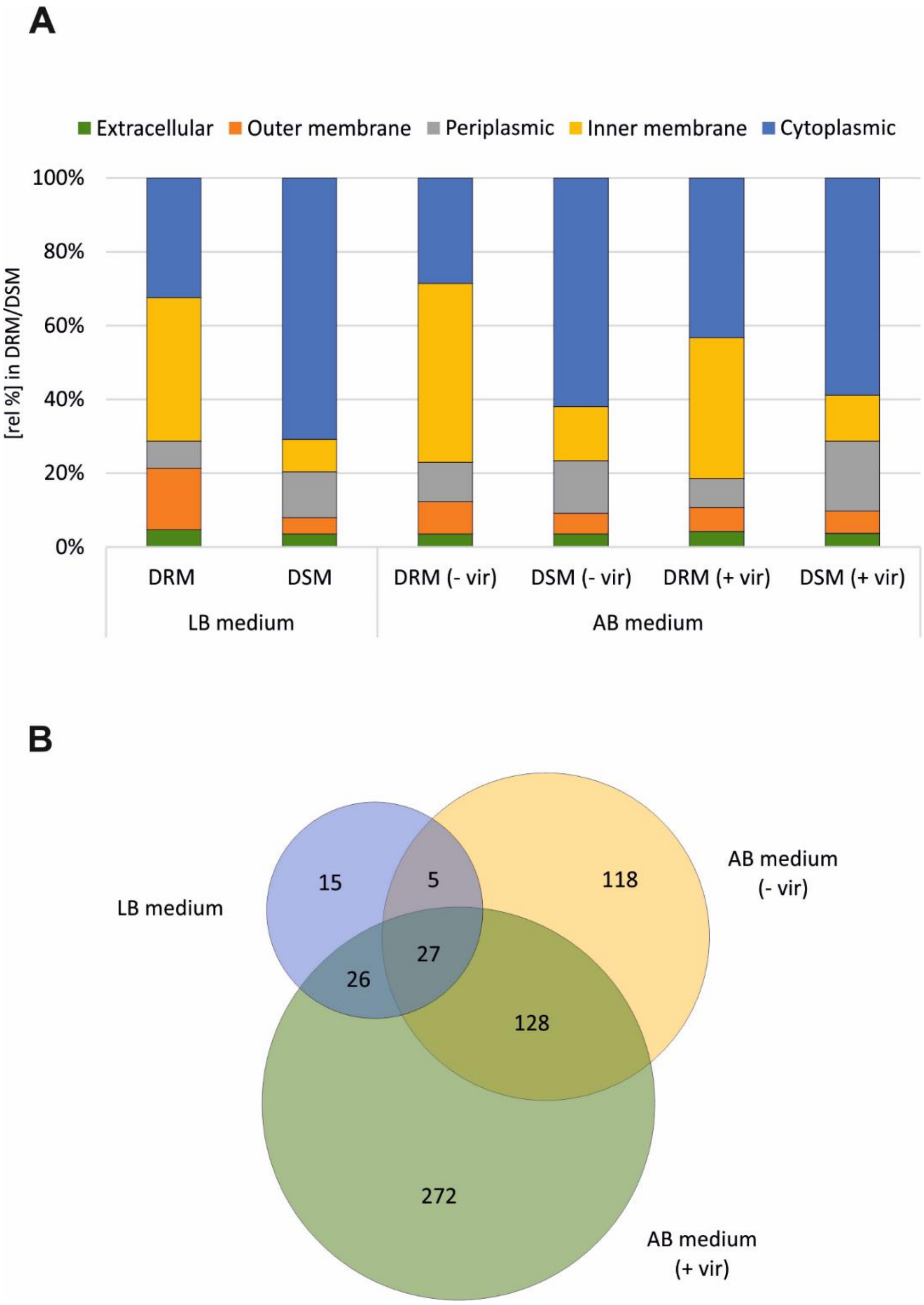

Fig. S2: Prediction of the cellular localization of proteins found enriched in DRMs or DSMs under different growth conditions. (A) The cellular localization was predicted using the CELLO2GO webserver (Yu et al., 2014). – vir: non-induced cultures; + vir: virulence-induced cultures. (B) Venn diagram showing the overlap of proteins identified in DRMs under the different culture conditions.

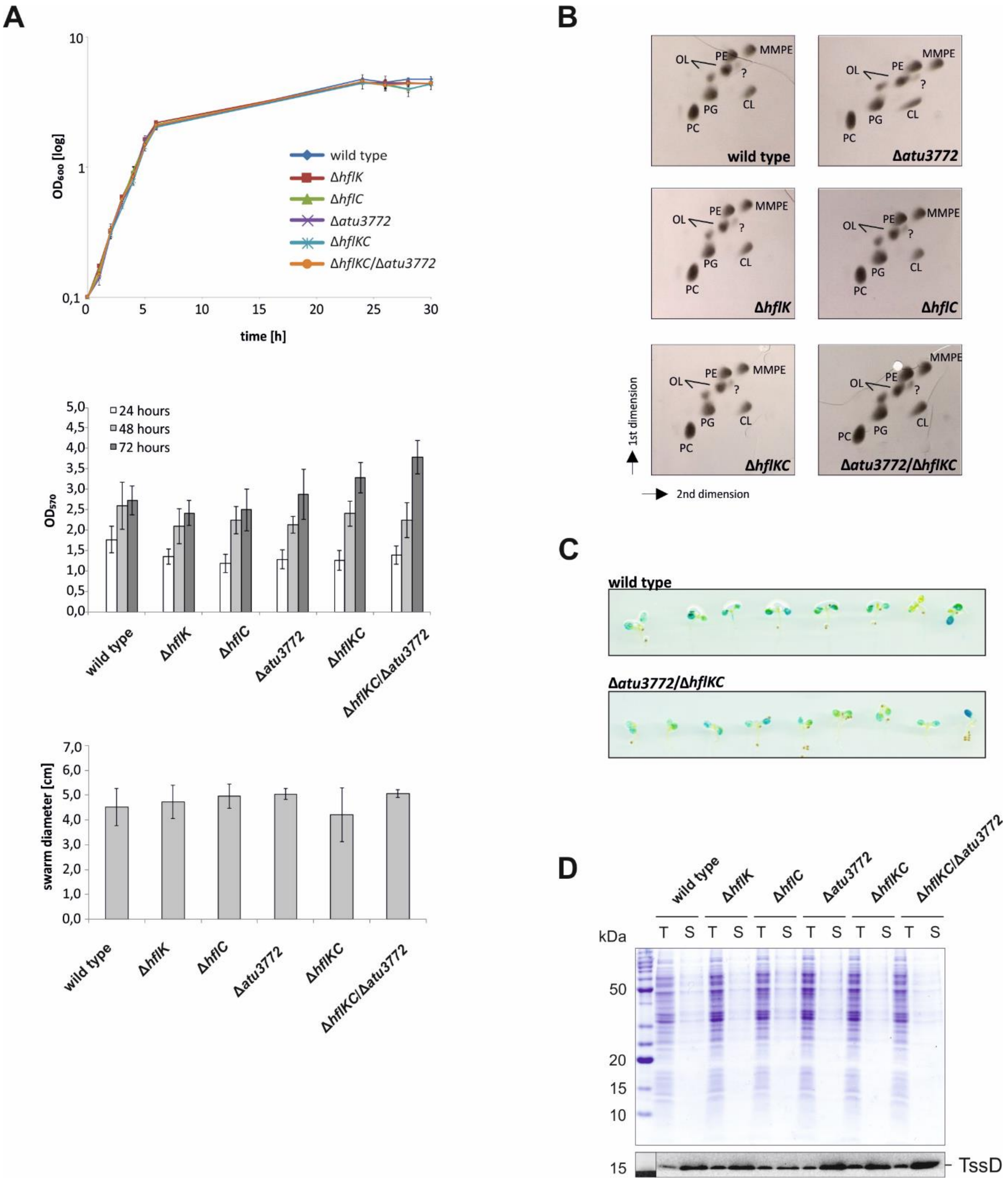

Fig. S3: Phenotypic characterization of SPFH deletion strains. (A) Cellular growth in LB medium was monitored for 30 h and OD_{600} values were plotted against time (upper panel). Biofilm formation in microtiter plates was analyzed by staining biomass with crystal violet. Adherent biomass was quantified at indicated time points by measuring the absorbance of solubilized dye in supernatants at 570 nm (middle panel). Note that the Δ*atu3772*/Δ*hflKC* strain produced slightly more biomass compared to the wild type, although the difference was not always statistically significant. Motility was analyzed by measurement of the swarm diameter of each strain after 24 h incubation on semi-solid

M9 agar (lower panel). Representative data from at least two independent biological replicates are shown. (B) Lipid profile of wild type and SPFH deletion strains. Lipids were isolated and separated via two-dimensional thin-layer chromatography according to standard protocols. PE: phosphatidylethanolamine; MMPE: monomethyl-PE; OL: ornithine lipid; PG: phosphatidylglycerol; PC: phosphatidylcholine; CL: cardiolipin. Unknown lipids are indicated by question mark. (C) GUS histochemical analysis of *Arabidopsis* seedlings infected with *A. tumefaciens* wild type (Wt) and the SPFH triple mutant (Δ*atu3772*/Δ*hflKC*). GUS staining (blue leaves) indicates *Agrobacterium*-mediated DNA transfer via T4SS and was not altered in the mutant strain. (D) TssD is detected in total cells (T) and secreted to culture supernatants (S) via T6SS-depentent secretion in the wild type and all SPFH mutant strains.

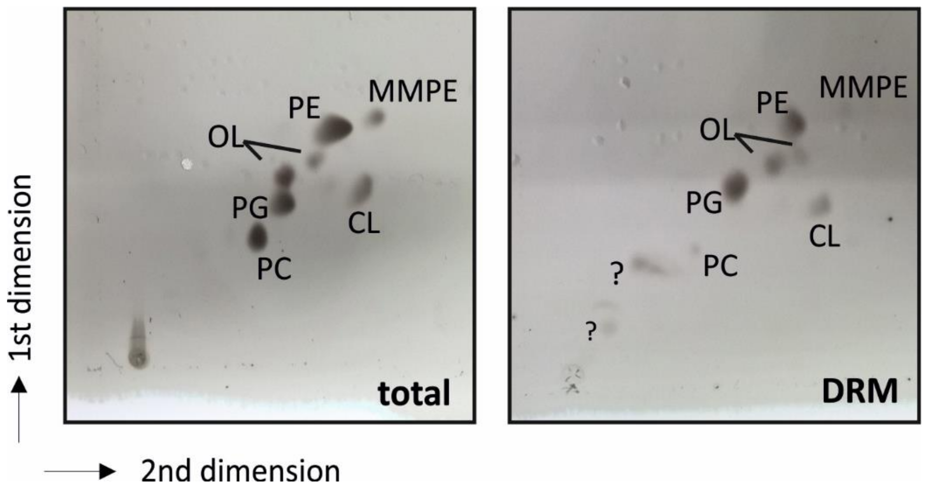

Fig. S4: Lipid profile of total cells and DRMs after growth in rich medium. Lipids were isolated and separated via two-dimensional thin-layer chromatography according to standard protocols (Czolkoss et al., 2016). Lipids were identified by comparing their migration characteristics to commercially available standards. PE: phosphatidylethanolamine; MMPE: monomethyl-PE; OL: ornithine lipid; PG: phosphatidylglycerol; PC: phosphatidylcholine; CL: cardiolipin. Unknown lipids are indicated by question mark.

References

Czolkoss, S., Fritz, C., Hölzl, G., and Aktas, M. (2016). Two distinct cardiolipin synthases operate in *Agrobacterium tumefaciens*. *PLoS One* 11(7)**,** e0160373. doi: 10.1371/journal.pone.0160373.

Hanahan, D. (1983). Studies on transformation of *Escherichia coli* with plasmids. *J Mol Biol* 166(4)**,** 557-580. doi: 10.1016/s0022-2836(83)80284-8.

Hoffmann, M.C., Wagner, E., Langklotz, S., Pfänder, Y., Hott, S., Bandow, J.E., et al. (2015). Proteome profiling of the *Rhodobacter capsulatus* molybdenum response reveals a role of IscN in nitrogen fixation by Fe-Nitrogenase. *J Bacteriol* 198(4)**,** 633-643. doi: 10.1128/JB.00750-15.

Knoke, L.R., Abad Herrera, S., Götz, K., Justesen, B.H., Günther Pomorski, T., Fritz, C., et al. (2020). *Agrobacterium tumefaciens* Small Lipoprotein Atu8019 Is Involved in Selective Outer Membrane Vesicle (OMV) Docking to Bacterial Cells. *Front Microbiol* 11**,** 1228. doi: 10.3389/fmicb.2020.01228.

Möller, P., Overlöper, A., Förstner, K.U., Wen, T.N., Sharma, C.M., Lai, E.M., et al. (2014). Profound impact of Hfq on nutrient acquisition, metabolism and motility in the plant pathogen *Agrobacterium tumefaciens*. *PLoS One* 9(10)**,** e110427. doi: 10.1371/journal.pone.0110427.

Schäfer, A., Tauch, A., Jager, W., Kalinowski, J., Thierbach, G., and Pühler, A. (1994). Small mobilizable multi-purpose cloning vectors derived from the *Escherichia coli* plasmids pK18 and pK19: selection of defined deletions in the chromosome of *Corynebacterium glutamicum*. *Gene* 145(1)**,** 69-73. doi: 10.1016/0378-1119(94)90324-7.

Yu, C.S., Cheng, C.W., Su, W.C., Chang, K.C., Huang, S.W., Hwang, J.K., et al. (2014). CELLO2GO: a web server for protein subCELlular LOcalization prediction with functional gene ontology annotation. *PLoS One* 9(6)**,** e99368. doi: 10.1371/journal.pone.0099368.
